# The Use of Bright and Dark Types of Humour is Rooted in the Brain

**DOI:** 10.1038/srep42967

**Published:** 2017-02-17

**Authors:** Ilona Papousek, Willibald Ruch, Christian Rominger, Elisabeth Kindermann, Katharina Scheidl, Günter Schulter, Andreas Fink, Elisabeth M. Weiss

**Affiliations:** 1 University of Graz, Department of Psychology, Biological Psychology Unit, Graz, Austria; 2 University of Zurich, Department of Psychology, Zurich, Switzerland

## Abstract

The ways in which humour can be used are related to the manifold interpersonal functions humour can serve, some of which are positive, and some negative. In the present study, phasic changes in the functional coupling of prefrontal and posterior cortex (EEG coherence) during other people’s auditory displays of happy and sad mood were recorded to predict people’s typical use of humour in social interactions. Greater use of benevolent humour, the intentions of which are in keeping with the characteristics of “laughing-with” humour, was associated with greater decreases of prefrontal-posterior coupling during the processing of happy laughter. More loose prefrontal-posterior coupling indicates loosening of control of the prefrontal cortex over the incoming perceptual information, thereby opening up the perceptual gate and allowing the brain to become more affected by the social-emotional signals. Greater use of humour styles linked to malicious intentions of “laughing-at” humour was associated with responses indicating a wider opened perceptual gate during the processing of other people’s crying. The findings are consistent with the idea that typical humour styles develop in line with the rewarding values of their outcomes (e.g., interaction partners are happy or hurt), which in turn are defined through the individuals’ latent interpersonal goals.

The use of humour is a common component in social interaction, where it can serve different purposes. A common distinction, also confirmed by psychometric analysis, is that between using humour with the intention to laugh *with* others versus the intention to laugh *at* others^1^. The ways in which humour can be used are related to the manifold interpersonal functions humour can serve, some of which are positive, and some negative^2^. Shared humour (laughing with somebody else) is an important social bonding mechanism, it aids the formation, enhancement, and maintenance of social relationships, and enhances feelings of connectedness and closeness^3^,^4^,^5^,^6^,^7^,^8^,^9^,^10^,^11^. Interpersonal functions of laughing at others include manipulative control, status enhancement or maintenance, ostracism of out-group members, and enforcement of conformity^12^,^13^,^14^.

If the interpersonal intentions coupled with the ways in which humour can be used are not limited to the moment but are manifested as a trait and, thus, are mirrored in the typical use of certain types of humour, the latter may be rooted in relevant social-emotional brain functions. For the following reasons, the brain’s automatic responses to the perception of other people’s displays of happy and depressed or despaired emotional states seemed to be a promising candidate for finding neurological roots of bright and dark humour styles.

Humour styles refer to the ways in which humour is typically used in social interactions, and there are multiple ways to categorise them^15^. We used a classification in the present study that specifically focuses on the use/production of humour (as opposed to the appreciation of humour produced by others) and includes the attempt to define the goals and intentions of the use of humour paired with the attitudes towards the targets. The classification goes back to Schmidt-Hidding^16^, who initially used a lexical approach and analysed humour in literature. The eight humour styles identified by Schmidt-Hidding were picked up by Ruch who analyzed them from a psychological perspective and cast them in a psychometrically sound self-report instrument^15^,^17^,^18^. Factor analysis of the structure of these eight humour styles suggested a clear cluster referring to “laughing-at” styles comprising cynicism, sarcasm, and irony, one factor comprising only benevolent humour (“laughing with”), and one separate factor comprising fun (clowning around) and nonsense humour. Wit and satire had double loadings and, thus, can apparently be used in negative as well as positive ways^17^,^18^. The empirical clusters largely match the social goals that Schmidt-Hidding had attributed to the humour styles: Malicious, mean-spirited goals and attitudes, intentions of hurting other persons and demonstrating superiority are attributes of cynicism, sarcasm, irony, in part also of satire and wit. To brighten others up and point up funny sides of adversities or short-comings in order to make others laugh about them are goals of the benevolent humour style. Schmidt-Hidding did not attribute significant social goals to the styles nonsense and fun. The set of humour styles overlaps with previous ones (e.g., 8) but is more extensive^15^.

Broadly defined, goals refer to internal representations of desired states^19^. The goals attributed to the humour styles most probably refer to “latent” goals^19^, which can motivate action and direct behaviour outside of people’s awareness^20^ and which play key roles in many aspects of social life (e.g., moral behaviour, social discrimination^21^). It is believed that positive reward signals are attached to the outcomes of goals via the mesolimbic dopamine system^20^,^22^,^23^. Whether a goal is pursued depends on its rewarding value, that is, on the extent to which the outcome is desired and rewarding^19^.

Considering an interpersonal view of the desired outcomes, the goals attached to the humour styles may be summarised as either to make the targets happy and laugh (“laughing-with” type of humour), or to hurt the targets, make them cry (“laughing-at” styles). Taken together, this means that the respective outcomes (social interaction partners are happy or hurt) and, as a consequence thereof, the respective displays of happy/exhilarated (laughter) or depressed/despaired (crying) emotional states have a desired, rewarding value.

Pursuing this train of thought, one may expect different brain responses in individuals with lesser versus greater use of bright (“laughing-with”) or dark (“laughing-at”) types of humour when they are exposed to other people’s laughter and crying, depending on the supposed rewarding value of the stimulus. Specifically, this concerns phasic changes in the functional coupling of prefrontal and posterior association cortex, measured by changes of EEG coherence, which signify modulation of incoming affectively laden social information. More loose prefrontal-posterior coupling during social-emotional processing, especially in the right hemisphere, indicates loosening of control of the prefrontal cortex over the incoming perceptual information, thereby opening up the perceptual gate and allowing the brain to become more affected by the social-emotional signals. By contrast, functional coupling increases during exposure to aversive information, protecting the individual from being unduly affected by the aversive input^24^,^25^,^26^,^27^,^28^,^29^.

It may be expected, therefore, that typical use of “laughing-with” humour will be correlated with decreases of prefrontal-posterior coupling (EEG coherence) during the processing of other people’s happy laughter, allowing the brain to become more affected by this rewarding social-emotional signal. On the other hand, typical use of dark, “laughing-at” humour may be correlated with relative decreases of prefrontal-posterior coupling during the processing of other people’s crying.

However, there is at least one other possible outcome. Dark humour styles were associated with low interpersonal competence, particularly with poor ability to perceive other people’s emotions^30^,^31^,^32^. Therefore, it is possible that individuals poor in the perception of emotions tend to use humour in compromising ways, because they do not (appropriately) interprete the target’s emotional feedback. In regard to the brain responses, during the exposure to social-emotional signals, prefrontal-posterior coupling was also higher (gate more closed) in individuals with a generally lower propensity for perceiving the emotional states of other persons^26^. Consequently, if poorer emotion perception is the more crucial process underlying strong tendencies to use dark humour, use of dark humour styles may be expected to be associated with increases, rather than decreases, of prefrontal-posterior coupling in response to other people’s crying.

## Results

Standard multiple regression analyses were performed to examine whether individual differences in changes of prefrontal-posterior coupling during the perception of other people’s laughter or crying may predict the typical use of each of the eight types of humour captured by the 8SHCS. Change-of-coherence scores (Δcoh) during listening to the laughter and crying stimuli were used as the predictors. Because of the wide age range in the sample, and because brain connectivity^33^,^34^ as well as humour preferences^35^,^36^ may change with aging, age was included as an additional predictor to control for its potential influence.

Results of the effects of the change-of-coherence scores (Δcoh) in the right hemisphere during listening to other people’s laughter and crying are summarised in Table 1. The regression models were significant for cynicism (*F*(3,48) = 2.8, *p* = 0.049, R^2^ = 0.15), sarcasm (*F*(3,48) = 3.3, *p* = 0.027, R^2^ = 0.17), irony (*F*(3,48) = 4.0, *p* = 0.013, R^2^ = 0.20), wit (*F*(3,48) = 2.9, *p* = 0.045, R^2^ = 0.15), nonsense (*F*(3,48) = 3.8, *p* = 0.017, R^2^ = 0.19), and benevolent humour (*F*(3,48) = 3.3, *p* = 0.029, R^2^ = 0.17), and were not significant for satire (*F*(3,48) = 2.0, *p* = 0.132, R^2^ = 0.11) and fun (*F*(3,48) = 0.8, *p* = 0.494, R^2^ = .05; *α* = *0.05*). With the use of a p < 0.005 criterion for Mahalanobis distance no outliers among the cases were found.

The detailed results of the regression analyses depicted in Table 1 show that greater decreases of Δcoh during the perception of other people’s crying, that indicate a relatively more opened perceptual gate during this stimulus, were associated with a greater propensity to use cynical, sarcastic, and ironic humour, and wit. In contrast, greater decreases of Δcoh during the perception of other people’s laughter were associated with a greater propensity to use benevolent humour. Changes in prefrontal-posterior coupling during the laughter or the crying stimulus did not correlate with the use of satire, fun (clowning around), and nonsense humour. The semi-partial correlations denote the correlations between Δcoh during one social-emotional stimulus and use of a humour style, adjusted for Δcoh during the other stimulus and age. Table 1 shows that the semi-partial correlations remained virtually unchanged compared to the respective zero-order correlations. This suggests that the relationships between changes of prefrontal-posterior coupling and the use of humour were present independently from eventual correlations with age, and also that the responses to the two stimuli were largely independent. Additionally, the analyses revealed that, independently from the brain responses to the social-emotional stimuli, older participants indicated less use of sarcastic, ironic, and nonsense humour.

The pattern observed in these results corroborates the findings of the factor analysis in a larger sample which clearly yielded a factor comprising cynicism, sarcasm, and irony, and another factor comprising only benevolent humour^17^. Wit did not unequivocally load on either of these factors. It therefore seems that the correlations between the use of cynical, sarcastic, and ironic humour with the brain responses to other people’s crying are due to a common underlying characteristic of these humour styles.

**Figure 1:**
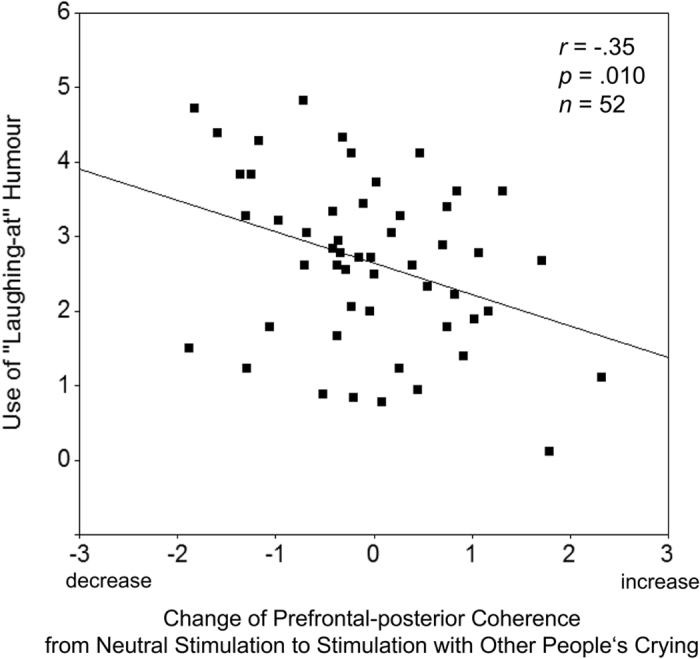
Correlation between changes of prefrontal-posterior EEG coherence in response to the perception of other people’s crying and use of dark (“laughing-at”) humour. Coherence changes in the right hemisphere (beta frequency range) relative to neutral stimulation. The plot shows standardised residuals (Δcoh; see Methods section). Negative scores of Δcoh denote a relative decrease of prefrontal-posterior coherence (more opened perceptual gate), positive scores denote an increase (gate more closed). “Laughing-at” humour comprises the styles cynicism, sarcasm, and irony.

**Figure 2:**
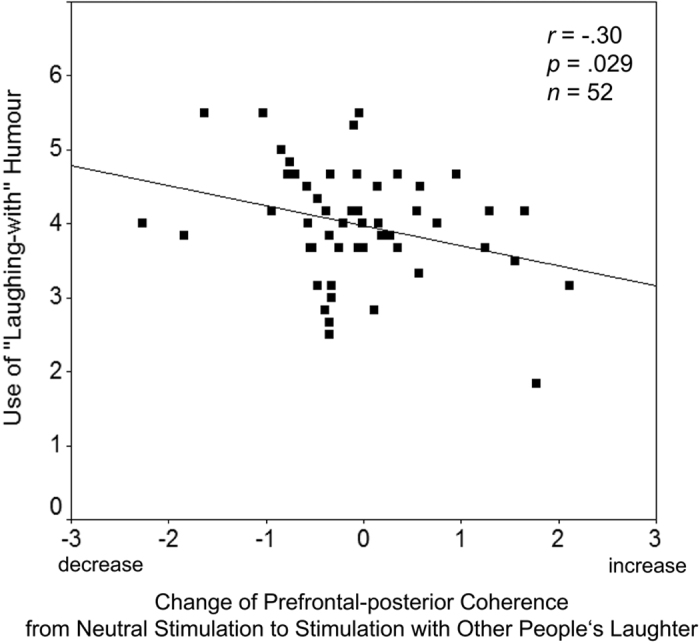
Correlation between changes of prefrontal-posterior EEG coherence in response to the perception of other people’s laughter and use of benevolent (“laughing-with”) humour. Coherence changes in the right hemisphere (beta frequency range) relative to neutral stimulation. The plot shows standardised residuals (Δcoh; see Methods section). Negative scores of Δcoh denote a relative decrease of prefrontal-posterior coherence (more opened perceptual gate), positive scores denote an increase (gate more closed).

The regression analyses demonstrate that the individuals’ use of “laughing-at” or “laughing-with” humour were specifically predicted by the changes of prefrontal-posterior coupling in response to other people’s crying or laughter, respectively. Additionally, two supplemental regression analyses were conducted to assess the correlations between the use of dark humour styles (i.e. averaged across cynicism, sarcasm, and irony) and benevolent humour, and Δcoh during the crying or the laughter stimulus. Results of these analyses are summarised in Table 2. The semipartial correlations show that decreases in prefrontal-posterior coupling during the crying stimulus were specifically correlated with the use of “laughing-at” humour but not with the use of “laughing-with” humour. Conversely, decreases in prefrontal-posterior coupling during the laughter stimulus were specifically correlated with the use of “laughing-with” humour but not with the use of “laughing-at” humour. Figures 1 and 2 show scatter plots of these correlations. The correlation between the use of “laughing-at” and “laughing-with” humour was *r* = 0.40 (*p* = 0.003, two-tailed), reflecting an additional superordinate factor of humourousness^17^.

Analogous regression analyses for the left hemisphere did not reveal significant correlations between prefrontal-posterior coherence changes and humour styles (highest *sr* = −0.12, *p* = 0.406). Use of the humour styles did not differ between men and women (in independent *t*-Tests *t*-values ranged from *t*(50) = 0.2, *p* = 0.866 to *t*(50) = 1.7, *p* = 0.105), and did not depend on the educational level of the participants (less then high school vs high school graduate or university; *t*-values ranged from *t*(50) = 0.5, *p* = 0.655 to *t*(50) = 1.8, *p* = 0.076), except for nonsense humour (greater use with higher educational levels, *M* = 4.2, *SD* = 1.2; *M* = 3.4, *SD* = 0.9; *t*(50) = 2.9, *p* = .006, η^2^ = 0.14).

## Discussion

The study showed that an individual’s typical use of bright (“laughing-with”) and dark (“laughing-at”) humour is linked to the brain’s automatic responses to incoming social-emotional information. Greater typical use of benevolent humour, the goals and intentions of which are in keeping with the characteristics of “laughing-with” humour, was associated with greater relative decreases of prefrontal-posterior coupling during the processing of other people’s happy laughter, allowing the brain to become more affected by this apparently rewarding social-emotional signal^24^,^25^,^26^,^27^,^28^,^29^. Greater typical use of the three humour styles that were consistent with malicious intentions of “laughing-at” humour (cynicism, sarcasm, irony^17^) was associated with greater relative decreases of prefrontal-posterior coupling (i.e., a wider opened perceptual gate) during the processing of other people’s crying. This finding is congruent with the assumed rewarding value of this social-emotional signal in people with a high propensity to use “laughing-at” kinds of humour. Use of nonsense humour and fun (clowning around), to which Schmidt-Hidding^16^ had not attributed significant social goals, and which constituted a separate factor (i.e., separate from typical “laughing-at” and “laughing-with” kinds of humour) in the large-sample study by Ruch^17^, was not related to the brain responses to other people’s affect expressions. It seems noteworthy, in this context, that benevolent humour may involve moral goodness or virtue^37^, which is lacking in sheer fun.

However, also one of the two more ambivalent types of humour (wit)^17^ correlated with decreases of prefrontal-posterior coupling during the crying stimulus. This might be explained by the nature of typical jokes, which almost always are made at someone else’s expense^38^. Thus, some malicious intentions are included in this type of humour, which in the current sample perhaps might have outweighed the benevolent parts.

Taken together, the findings do not support the alternative possibility that poor perception of other people’s emotions may be the most decisive factor predicting a high propensity for using “laughing-at” types of humour, despite the fact that dark humour styles were associated with low interpersonal competence^30^,^31^,^32^. If poor perception of other’s emotions had been more important, greater tendencies to use “laughing-at” humour would have been correlated with increased prefrontal-posterior coupling during the confrontation with other people’s crying (i.e., a more closed perceptual gate^26^).

It has been clearly recognized that the social effect of joking and laughing with somebody else, that is, the happy laughter of interacting partners, is an immediately pleasurable and rewarding experience for most people who produce humour^6^,^7^,^8^,^9^,^10^,^11^. The current findings and their theoretical foundation suggest that in some people (i.e., in those with a high propensity to use “laughing-at” humour), expressions of hurt, depressed or despaired feelings may have a rewarding value. Laughing at or ridiculing another person is an expression of disapproval that induces strong negative feelings in that person such as shame, feelings of inferiority and devaluation, which are very painful emotions^39^ and, therefore, have marked effects on the person’s outward emotional expression. There is, in fact, evidence that observing or causing other people’s adversity can be a source of pleasure^40^, a feature that is associated with psychopathic personality traits^41^. In line with that, several studies have shown relationships between the propensity of laughing at others and psychopathic personality traits such as antagonism, manipulation and callousness^14^,^42^,^43^,^44^. Previous research indicated that the use of dark types of humour may be one factor perpetuating maladaptive cognitive schemas that implicate the belief that one is superior to others and that others should be controlled and dominated^45^. These maladaptive cognitive schemas are also associated with vulnerability to development of externalising/aggressive psychopathology^46^,^47^,^48^.

Apart from potentially maladaptive features and developments in those with high tendencies to use humour in dark ways, using humour with a view to laughing at other people can have drastic negative social consequences. For instance, in line with the interpersonal functions, goals, and desired outcomes that are attached to “laughing-at” types of humour, it was shown that, from a very early age on (6 years) a high propensity to enjoy laughing at others was related to bullying behaviour^49^, which can entail devastating effects on the victims and their social behaviour^50^,^51^. On the other hand, there is evidence that the use of benevolent humour targeted to laugh with somebody else may help to protect against the development of depression^52^.

Collectively, the pattern of the present findings is largely consistent with the idea that an individual’s typical humour style develops in line with the rewarding values of its outcomes (e.g., social interaction partners are happy or hurt), which in turn are defined through the – latent – interpersonal goals of the individual. That way, the typical use of bright and dark types of humour seems to be rooted in the brain, and hence may provide indications of biologically anchored clinically and socially relevant personality features.

## Methods

### Participants

A total of n = 52 participants (21 men, 31 women) completed the experiment (age range 20 to 71 years, *M* = 36.7, *SD* = 14.4). Levels of education were: less than high school (29), high school graduate (19), university degree (4). All participants were right-handed as confirmed by a standardised hand skill test. Individuals who reported having a neuropsychiatric disease or using psychoactive medication were not included in the study. Participants were requested to refrain from alcohol for twelve hours and from coffee and other stimulating beverages for two hours prior to their lab appointment, and to come to the session well rested. The study was performed in accordance with the American Psychological Association’s Ethics Code and the Declaration of Helsinki and was approved by the ethics committee of the University of Graz. Informed consent was obtained from all participants.

### Assessment of Humour Styles

In the 8SHCS (8 Schmidt-Hidding Comic Styles^18^), participants are asked to rate the extent to which 48 statements apply to the way they typically express humour on a seven-point Likert scale (from 0 – “strongly disagree” to 6 – “strongly agree”). The questionnaire comprises eight subscales (6 items each) capturing the propensity to use humour in the form of sarcasm (e.g., “Biting mockery suits me”, test reliability was *α* = 0.87 in the present study, *M* = 2.22, *SD* = 1.31), cynicism (e.g., “I tend to show no reverence for certain moral concepts and ideals, but only scorn and derision”, *α* = 0.89, *M* = 2.52, *SD* = 1.31), irony (e.g., “My irony unveils who is smart enough and understands something and who does not”, *α* = 0.80, *M* = 3.25, *SD* = 1.11), satire (“I like to ridicule moral badness to induce or increase a critical attitude in other people”, *α* = 0.81, *M* = 2.89, *SD* = 1.15), wit (e.g. “I surprise others with funny remarks and accurate judgements of current issues, which occur to me spontaneously”, *α* = 0.88, *M* = 3.48, *SD* = 1.08), benevolent humour (e.g., “When my humour is aimed at human weaknesses, I include both myself and others”, *α* = 0.69, *M* = 3.99, SD = 0.77), fun (e.g., “I like to make jests and to be silly”, *α* = 0.89, *M* = 3.53, *SD* = 1.30), and nonsense (e.g., “Humour doesn’t have to make sense; the opposite holds true for me: the more absurd, the funnier”, *α* = 0.83, *M* = 3.76, *SD* = 1.11). Self-peer correlations were between *r* = 0.40 and *r* = 0.56 with a median of *r* = 0.49^18^.

### Social-emotional Stimulation

Three sound recordings in which a small mixed-gender group of people audibly expressed the respective affect without using language (words or parts of words) were used (90 s each): Laughter (good-natured, hearty laughter), Crying (bitter crying and sobbing), and a neutral recording (soft murmurs and trivial everyday sounds without understandable language), serving as the reference condition. The clips were matched for peak sound intensity and sound level range, and were presented over headphones. They have been used in several previous studies^25^,^26^,^27^,^28^,^29^. The displayed emotions are unambiguous and intense; healthy participants have no difficulties identifying and differentiating the expressed affective states^25^,^29^.

### EEG Recording and Quantification

The EEG was recorded using a Brainvision BrainAmp Research Amplifier (Brain Products, sampling rate of 500 Hz, resolution 0.1 μV), referenced to the nose and re-referenced offline to a mathematically averaged ears refs 53, 54. All data were inspected visually, in order to eliminate intervals in which ocular or muscle artefacts occurred. At least 30 s of artefact free data was obtained for each participants for each of the recording periods and for each of the electrode positions of interest. Artefact-free EEG data were submitted to Fast Fourier Analysis using a Hanning window (epoch length 1 s, overlapping 50%), and spectral coherence (Fisher’s z-transformed) was obtained. Previous research on EEG coherence in the context of affective processing indicated that connectivity changes during evoked emotions occurred primarily in the beta frequency range^24^,^25^,^26^,^27^,^28^,^29^,^55^,^56^,^57^. Consequently, we focused on coherence in the beta range (13–30 Hz).

Following previous relevant research^24^,^25^,^26^,^27^,^28^,^29^,^56^, coherence pairs were grouped into anatomically valid clusters corresponding to the left and right, prefrontal and posterior association cortex regions. Coherence scores of nine electrode pairs each were averaged to summarise interaction within the left and the right hemisphere, respectively (left: Fp1-T3, Fp1-P3, Fp1-T5, F3-T3, F3-P3, F3-T5, F7-T3, F7-P3, F7-T5; right: Fp2-T4, Fp2-P4, Fp2-T6, F4-T4, F4-P4, F4-T6, F8-T4, F8-P4, F8-T6).

Linear regressions were conducted using the EEG beta coherence during the neutral (reference) stimulus preceding the emotional stimulus to predict the coherence during listening to each of the emotional sound clips, in order to calculate residualised change scores (cf.^25^,^26^,^27^,^28^,^29^,^56^). These were used as indexes of state-dependent relative decreases or increases of intra-hemispheric coherence in response to the social-emotional stimulation. This was done to ensure that the analysed residual variability was due to the experimental manipulation, and not to individual differences in baseline levels, and to control for measurement error inherent in the use of repeated measures of the same kind^58^,^59^. The abbreviation “Δcoh” is used for these change-of-coherence scores. Negative scores indicate a relative decrease in prefrontal-posterior coherence, positive scores indicate a relative increase.

Since previous research indicated strong right-hemisphere dominated effects of coherence changes in the context of emotional processing, the analysis focused on prefrontal-posterior coherence changes in the right hemisphere, and a separate (supplemental) set of regression analyses tested for potential effects in the left hemisphere (cf.^25^,^26^,^27^,^28^,^29^).

### Procedure

After completing the handedness test, participants were seated in an acoustically and electrically shielded examination room, and electrodes were attached. They were instructed to close their eyes, to direct their whole attention to the sound recordings, and to imagine that they were amidst the happenings. A two-minutes resting period preceded the stimulation. The order of emotional sounds was counterbalanced, and emotional and neutral sound clips were presented in alternating order, so that each emotional sound was preceded by the neutral sound (i.e., N-S-N-C or N-C-N-S). Before each sound clip, the instructions were briefly repeated. The 8SHCS was completed in a separate test session.

## Additional Information

**How to cite this article**: Papousek, I. *et al*. The Use of Bright and Dark Types of Humour is Rooted in the Brain. *Sci. Rep.* **7**, 42967; doi: 10.1038/srep42967 (2017).

**Publisher's note:** Springer Nature remains neutral with regard to jurisdictional claims in published maps and institutional affiliations.

## Figures and Tables

**Table 1:** Prediction of use of types of humour by brain responses to other people’s laughter and crying (changes of prefrontal-posterior EEG coherences, Δcoh).

	Δcoh (laughter)		Δcoh (crying)		Age	
	*r (p)*	*sr (p)*	*r (p)*	*sr (p)*	*r (p)*	*sr (p)*
Cynicism*	−0.05 (0.712)	−0.07 (0.600)	**−0.33 (0.018)**	**−0.31 (0.024)**	−0.23 (0.104)	−0.19 (0.156)
Sarcasm*	0.05 (0.719)	0.04 (0.774)	**−0.31 (0.027)**	**−0.27 (0.044)**	**−0.31 (0.027)**	**−0.28 (0.040)**
Irony*	−0.10 (0.483)	−0.11 (0.384)	**−0.32 (0.021)**	**−0.30 (0.027)**	**−0.32 (0.020)**	**−0.29 (0.032)**
Satire	−0.20 (0.157)	−0.22 (0.113)	−0.23 (0.098)	−0.26 (0.067)	0.06 (0.672)	0.09 (0.499)
Wit*	−0.08 (0.561)	−0.10 (0.445)	**−0.34 (0.014)**	**−0.33 (0.018)**	−0.20 (0.152)	−0.16 (0.226)
Fun	0.04 (0.781)	0.03 (0.839)	−0.19 (0.171)	−0.18 (0.212)	−0.12 (0.393)	−0.10 (0.472)
Nonsense*	0.08 (0.575)	0.09 (0.539)	−0.18 (0.216)	−0.13 (0.338)	**−0.41 (0.003)**	**−0.39 (0.004)**
Benevol. humour*	**−**0**.30 (0.029)**	**−0.31 (0.021)**	−0.21 (0.136)	−0.21 (0.110)	−0.19 (0.186)	−0.15 (0.247)

*Note:* *Statistically significant regression models (*F*-test). *r* = zero-order correlation, *sr* = semipartial correlation, *p* = *p*-value (two-tailed). Coherence changes in the right hemisphere (beta frequency range) relative to neutral stimulation. Negative scores of Δcoh denote a relative decrease of prefrontal-posterior coherence (more opened perceptual gate), positive scores denote an increase (gate more closed). Significant zero-order and semi-partial correlations are highlighted in bold font (α = 0.05). *N* = 52.

**Table 2:** Correlations of brain responses to other people’s laughter and crying (changes of prefrontal-posterior EEG coherences, Δcoh) with unique variance of the typical use of “laughing-at” and “laughing-with” humour.

	“laughing-at”		“laughing-with”	
	*r (p)*	*sr (p)*	*r (p)*	*sr (p)*
Δcoh (laughter)	−0.21 (0.136)	−0.07 (0.584)	**−0.30 (0.029)**	**−0.32 (0.024)**
Δcoh (crying)	**−0.35 (0.010)**	**−0.29 (0.033)**	−0.03 (0.815)	0.10 (0.476)

*Note: r* = zero-order correlation, *sr* = semipartial correlation, *p* = *p*-value (two-tailed). Coherence changes in the right hemisphere (beta frequency range) relative to neutral stimulation. Negative scores of Δcoh denote a relative decrease of prefrontal-posterior coherence (more opened perceptual gate), positive scores denote an increase (gate more closed). Significant zero-order and semi-partial correlations are highlighted in bold font (α = 0.05). These correlations are considered to be of medium size according to the common conventions of Cohen^60^. *N* = 52.
